# Age-Related EEG Features of Bursting Activity During Anesthetic-Induced Burst Suppression

**DOI:** 10.3389/fnsys.2020.599962

**Published:** 2020-12-03

**Authors:** Stephan Kratzer, Michael Schneider, David P. Obert, Gerhard Schneider, Paul S. García, Matthias Kreuzer

**Affiliations:** ^1^Department of Anesthesiology and Intensive Care Medicine, School of Medicine, Technical University Munich, Munich, Germany; ^2^Department of Anesthesiology, Columbia University, New York, NY, United States

**Keywords:** burst suppression, electroencephalography (EEG), elderly, general anesthesia, monitoring

## Abstract

Electroencephalographic (EEG) Burst Suppression (BSUPP) is a discontinuous pattern characterized by episodes of low voltage disrupted by bursts of cortical synaptic activity. It can occur while delivering high-dose anesthesia. Current research suggests an association between BSUPP and the occurrence of postoperative delirium in the post-anesthesia care unit (PACU) and beyond. We investigated burst micro-architecture to further understand how age influences the neurophysiology of this pharmacologically-induced state. We analyzed a subset of EEG recordings (*n* = 102) taken from a larger data set previously published. We selected the initial burst that followed a visually identified “silent second,” i.e., at least 1 s of iso-electricity of the EEG during propofol induction. We derived the (normalized) power spectral density [(n)PSD], the alpha band power, the maximum amplitude, the maximum slope of the EEG as well as the permutation entropy (PeEn) for the first 1.5 s of the initial burst of each patient. In the old patients >65 years, we observed significantly lower (*p* < 0.001) EEG power in the 1–15 Hz range. In general, their EEG contained a significantly higher amount of faster oscillations (>15 Hz). Alpha band power (*p* < 0.001), EEG amplitude (*p* = 0.001), and maximum EEG slope (*p* = 0.045) all significantly decreased with age, whereas PeEn increased (*p* = 0.008). Hence, we can describe an age-related change in features during EEG burst suppression. Sub-group analysis revealed no change in results based on pre-medication. These EEG changes add knowledge to the impact of age on cortical synaptic activity. In addition to a reduction in EEG amplitude, age-associated burst features can complicate the identification of excessive anesthetic administration in patients under general anesthesia. Knowledge of these neurophysiologic changes may not only improve anesthesia care through improved detection of burst suppression but might also provide insight into changes in neuronal network organization in patients at risk for age-related neurocognitive problems.

## Introduction

Electroencephalographic (EEG) recordings during the perioperative period can help assess a patient’s brain under anesthesia. The EEG activity changes from a high frequency/low amplitude pattern during wakefulness to low frequency/high amplitude waves during anesthesia (Brown et al., 2010). If exposed to even higher concentrations of the hypnotic agent (i.e., propofol or sevoflurane), the electrical activity of the brain switches from being dominated by slow and moderate frequency oscillations to a discontinuous state characterized by alternating episodes of cortical activity interrupted by epochs of voltage attenuation without oscillations. This state is often referred to as burst suppression (BSUPP; Swank and Watson, 1949). The neuronal mechanisms underlying periods of suppression likely involve activation of intrinsic inhibitory currents as well as a decrease in excitatory input to cortical neurons (Lukatch et al., 2005). It is still unclear whether cortical burst discharges are primarily driven by thalamocortical inputs or if neocortical burst suppression activity is intrinsically generated by the cortex itself (Lewis et al., 2013). Although the pattern may be associated with adverse neurocognitive patient outcomes, such as postoperative delirium or postoperative neurocognitive decline (Soehle et al., 2015; Fritz et al., 2016), anesthetic protocols designed to prevent BSUPP have failed to result in large changes in delivered anesthetic agent or on delirium outcomes. Hence, the controversy on the subject persists (Shortal et al., 2019; Wildes et al., 2019), as it does for the general relation between EEG-based monitoring and outcome (Berger et al., 2020; García, 2020). This may in part be related to the difficulty in identifying classic burst suppression patterns in vulnerable patients and because the surgical population most affected by postoperative neurocognitive disorders overlap with the population more likely to develop BSUPP. Purdon et al. described the increase in the occurrence of BSUPP with age (Purdon et al., 2015). To reliably identify this electroencephalographic feature a detailed description of the “burst” during BSUPP is crucial. EEG amplitude (total power) under general anesthesia decreases with age; complicating the interpretation of EEG signals (Schultz et al., 2004; Purdon et al., 2015; Kreuzer et al., 2020). Current commercial monitoring approaches mainly focus on the identification of the isoelectric episodes, i.e., they rather perform a “*suppression detection*” than a “*burst and suppression detection*” (Rampil, 1998; Särkelä et al., 2002; Jensen et al., 2014). These approaches may underestimate the real occurrence of burst suppression (Muhlhofer et al., 2017). A better understanding of burst features could help to supplement the suppression-based detection by adding information regarding the burst. To more closely investigate the age-induced changes in the EEG characteristics of BSUPP and especially on the bursts, we used data from a previously published study (Hesse et al., 2019). To ensure a quasi-steady-state condition we focused on the first burst, i.e., after the brain switched to BSUPP during induction with propofol. To investigate a possible impact of premedication with midazolam, we conducted a sub-group analysis. With our findings we can add to the existing knowledge regarding the effects of age on the EEG described for general anesthesia without BSUPP (Schultz et al., 2004; Purdon et al., 2015; Kreuzer et al., 2020), sleep (Carrier et al., 2001), and (relaxed) awake states (Polich, 1997).

## Materials and Methods

### Patients

For our analyses, we included 168 patients undergoing surgical intervention with general anesthesia that developed BSUPP during anesthesia induction with propofol that were recruited in the Atlanta hospitals for a previous study (Hesse et al., 2019).

**Figure 1:**
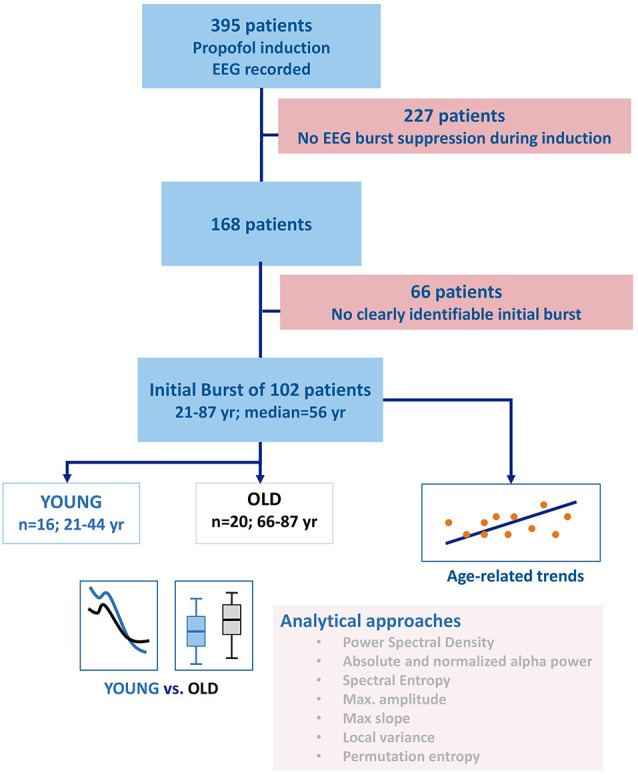
Flow chart describing patient and group selection as well as the analyses performed. After selecting the patients with clearly identifiable burst suppression, a set of spectral and time-domain parameters was used to investigate the influence of age on the electroencephalogram (EEG) during a burst. Besides the evaluation of the entire power spectrum, the parameters of choice were absolute and relative alpha band power, maximum amplitude, maximum slope, spectral entropy, permutation entropy, and the local variance of the EEG.

The study protocol was approved by local Ethics or Institutional Review Boards (Emory University). Written informed consent was obtained from each patient. In the original study, we only enrolled patients that we expected to be admitted to the post-anesthesia care unit (PACU) after non-emergency and non-cardiac surgery. We included 102 patients with a clearly identifiable initial burst. Sixteen patients younger than 45 years formed the YOUNG group for our investigation and 20 patients older than 65 years formed the OLD group. We arbitrarily chose the age thresholds for the YOUNG and OLD group but oriented ourselves at EEG related work that defined a patient being OLD if the age was more than 60 (Wildes et al., 2019) or 70 years of age (Purdon et al., 2015). The anesthesia teams were not required to follow any specific pharmacologic protocol for the induction or maintenance of general anesthesia. For this retrospective analysis, we examined the data from patient records collected at a single site who received propofol as an induction agent followed by sevoflurane for maintenance. The detailed information regarding the initial study is published by Hesse et al. (2019). We included patients that developed BSUPP during anesthesia induction with propofol. With our analyses, we focused on the EEG information and EEG changes in our patients that could help to improve EEG-based monitoring, but also impact monitoring by age-related EEG changes. Because 83 patients received midazolam as premedication we decided to additionally look at these cases separately and hence conducted a sub-analysis regarding a possible influence of midazolam on our results. Six of the 16 patients in the OLD group did not receive midazolam whereas every YOUNG patient had midazolam premedication. Figure 1 presents the flow chart of the selection and analysis process.

### EEG Recording

We recorded EEG from all patients using a SEDLine Legacy monitor (Masimo, Irvine, CA, USA) with an electrode strip placed on the forehead of the patient according to the manufacturer’s specification. The sample rate of the EEG was 250 Hz. Before burst extraction, we band-pass filtered the signal from 0.5 to 47 Hz with the MATLAB *filtfilt* function and downsampled the EEG to 125 Hz.

### Burst Extraction and Analysis

The identifying person was blinded to the age of the patient. The examiner visually identified the initial burst (longer than 1.5 s) in each recording. For analysis, the burst EEG had to follow at least 1 s of visually identified severe attenuation of EEG amplitude (<10 μV). The concept of the “silent second” has been previously described as a suitable concept to reliably detect the onset of EEG burst suppression (Pilge et al., 2014). Bursts can vary in duration (Lewis et al., 2013). To minimize the influence of temporal burst characteristics, i.e., the way the EEG characteristics changes within one burst over time, we refrained from analyzing the EEG trace beyond the first 1.5 s of each burst. Supplementary Figure 1 presents exemplary bursts from the young and old age group.

### Analyses of the Initial Burst Episodes

We performed all quantitative EEG analyses with MATLAB R2017a (MathWorks, Natick, MA, USA). For the selected 1.5 s, we calculated the power spectral density (PSD) using the *pwelch* function with a frequency resolution of 0.8 Hz. Because of the age-related change in EEG amplitude and hence spectral power (Schultz et al., 2004; Purdon et al., 2015), we also used the normalized PSD (nPSD), i.e., the PSD divided by the total power within the 0.8–30 Hz range reflecting the architecture of the EEG, for our analyses. To use one parameter that may serve as a proxy for processed EEG indices used for patient monitoring, we calculated the spectral entropy (SpEnt) as used in the state and response entropy of the Entropy Module (GE Healthcare, Helsinki, Finland; Viertio-Oja et al., 2004). The SpEnt presents the Shannon Entropy applied to the power spectrum and it evaluates the shape of the spectrum. The more uniformly the power is distributed among the frequencies, the higher the SpEnt. A general slowing of the EEG as observed in anesthesia will cause a shift towards higher power in the lower frequencies. This leads to a “less uniformly” distributed spectrum causing SpEnt to decrease. Besides the spectral approach, we also conducted analyses in the time domain. Therefore, we calculated the maximum amplitude for each burst as well as the maximum slope, i.e., the largest difference in amplitude between two sample points. As a parameter that analyzes the signal in the time domain, but can also be related to spectral EEG features, we applied the permutation entropy (PeEn; Bandt and Pompe, 2002). The PeEn quantifies the probability distribution of rank patterns of a certain length, here three data points. The higher the PeEn the more uniformly distributed are the probabilities and the more irregular is the analyzed signal. The PeEn with the used settings, i.e., embedding dimension *m* = 3 and time lag *τ* = 1 estimates the centroid of the power spectrum (Berger et al., 2017). We further calculated the local signal variance as used in a standard burst suppression detection algorithm based on signal variance (An et al., 2015). It also can be applied to short signal segments (Yan et al., 2012). We calculated the parameter *varBS* for the selected, 1.5 s burst sequence using 10 consecutive data points with a one-point shift. This means we constructed a vector containing the local variance for the initial bursts for each patient. From these vectors, we then used the 95th percentile values for statistical analysis. Taking the 95th percentile value instead of the maximum allows for correction against outliers caused by potential artifacts. In contrast to the algorithm presented by An et al. (2015), we did not perform the logical decision regarding burst or suppression.

### Statistical Analysis

To appropriately present the results, we used descriptive and inference statistics. For age-related changes, we calculated a linear model for all included patients using the MATLAB *fitlm* function. We obtained the regression curve and conducted a one-sample *t*-test to compare the slope coefficient of the model against a slope of zero. Based on the complementary nature of our analyses, we did not perform a correction for multiple comparisons but present the exact *p*-values and the 95% confidence intervals of the slope coefficient instead (McDonald, 2009). We further derived the correlation strength, i.e., the fit of the model by calculating the *R*^2^ value. Additionally we calculated the Spearman’s correlation coefficient using the MATLAB *rho = corr (X, Y, “type,” “Spearman”)* function. For easier interpretation of the data, we also calculated an effect size between the YOUNG (<45 years) and OLD (>65 years) patients. We present our results as medians with minimum and maximum values or for the PSD as median and the median absolute deviation. We calculated the area under the receiver operating curve (AUC) with 10k-fold bootstrapped 95% confidence intervals for parameter values to measure the strength of the effect of age, i.e., the separability between YOUNG and OLD, using the Measures of Effect Size (MES) toolbox in MATLAB (Hentschke and Stüttgen, 2011). In general, an AUC above 0.9 or below 0.1 seems to indicate an outstanding effect, an AUC above 0.8 or below 0.2 indicates an excellent effect, and an AUC above 0.7 or below 0.3 indicates an acceptable effect (Mandrekar, 2010). For comparison of the PSD and nPSD between the YOUNG and OLD, we used the AUC with 95% confidence intervals. We considered a difference as significant if for at least two neighboring frequencies the 95% confidence interval did not contain 0.5. This analysis was following previous analyses (Fleischmann et al., 2018; Kreuzer et al., 2020).

## Results

### Demographics

From the 408 cases in the Atlanta hospitals, we included 102 patients with visually identifiable initial bursts. Figure 1 shows the experimental setup of our analyses. The median age of the 102 included patients was 56 years (1st and 3rd quartile: 49 and 64 years). We included 67 male and 35 female patients. Their American Society of Anesthesiologists (ASA) physical status classification (Mayhew et al., 2019) was ASA 1 for four, ASA 2 for 47, ASA 3 for 48, and ASA 4 for three patients. We present the detailed distributions as Supplementary Figures 2A–D. We did not observe a slope significantly different from 0 for the fentanyl equivalents with age: fentanyl equivalents μgkg^−1^ = 2.41 + 0.02 * age (*p* = 0.383; *R*^2^ = 0.00). The corresponding plot is presented as a Supplementary Figure 3.

### Spectral Analyses

**Figure 2:**
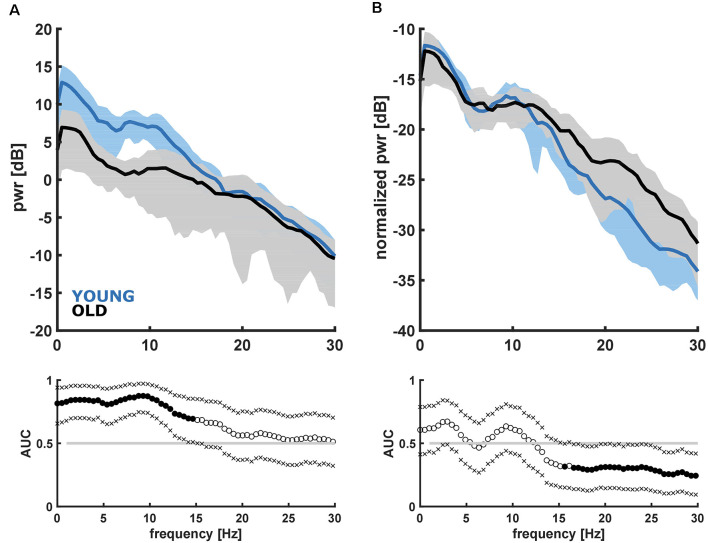
Absolute **(A)** and normalized **(B)** power spectral density and corresponding receiver operating curve (AUC) with 95% confidence intervals for the young (<45 years) and old (>65 years) patients. **(A)** YOUNG patients had significantly higher power in the frequencies up to 15 Hz. **(B)** OLD patients had significantly higher normalized power in the frequencies higher ~15 Hz.

Here we present the results from the linear models and the group comparisons between the young and the old patients. To better understand our results, we explicitly state YOUNG vs. OLD when we refer to group comparisons. When comparing the PSD of the YOUNG vs. OLD, we observed a higher power in the frequencies up to ~15 Hz in the YOUNG as depicted in Figure 2A. In the frequency range up to 15 Hz, the AUC was >0.7, and the 95% confidence intervals excluded 0.5. When focusing on the architecture of the EEG using the nPSD, we found significantly higher relative power (AUC >0.7 and 95% confidence intervals exclusive 0.5) in the bursts of the OLD starting at around 15 Hz as displayed in Figure 2B.

**Figure 3:**
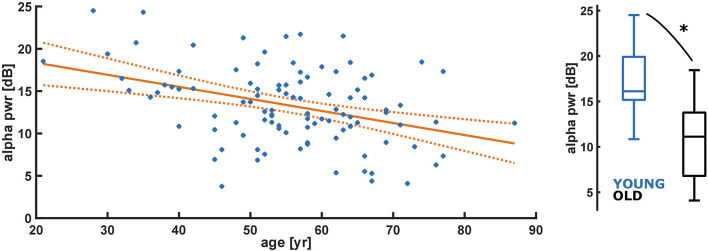
Linear regression model of decreasing absolute alpha band power (pwr) with age (year). Alpha power of the initial burst significantly (*p* < 0.001) decreases with age. Alpha pwr = −0.14*age + 21.20 (*p* < 0.001); YOUNG vs. OLD: AUC = 0.86 (0.73 0.97). *Indicates a significant difference (*p* < 0.05).

Evaluating the absolute alpha power over the entire age range, we found a significant decrease (*p* < 0.001) in alpha band power with age for the initial burst (Figure 3). However, normalized alpha power did not demonstrate an age effect (no significant difference from a slope of zero for the normalized alpha power, *p* = 0.719; Supplementary Figure 4A). Further, we did not find a significant effect for SpEnt (*p* = 0.145) as displayed in Supplementary Figure 4B. For the comparison between YOUNG and OLD including all patients we found an “excellent” effect of age on alpha power, i.e., lower alpha power in the OLD [AUC = 0.86 (0.73 0.97)]. We found no effect of age on a relative alpha power [AUC = 0.60 (0.40 0.78)] and SpEnt [AUC = 0.36 (0.18 50.55)] when comparing YOUNG and OLD. Table 1 presents the statistical parameters for the linear model of each parameter.

**Table 1:** Statistical parameters of the linear model (electroencephalographic, EEG parameter vs. age) and the comparisons between the YOUNG and OLD patients.

Linear model	Slope 95% CI	*p*	*t*-stat	*R*^2^	rho	YOUNG vs. OLD (AUC)
Alpha power = 21.20–0.14*age	−0.21, −0.07	<0.001	−4.11	0.14	−0.32	0.86 (0.73–0.97)
Norm. alpha power = −7.60–0.01*age	−0.07, 0.05	0.719	−0.36	0	−0.02	0.60 (0.40–0.78)
SpEnt = 2.90 + 0.01*age	−0.002, 0.012	0.145	1.47	0.02	0.14	0.36 (0.18–0.55)
Max amplitude = 31.97–0.26*age	−0.41, −0.10	0.001	−3.30	0.1	−0.35	0.84 (0.69–0.95)
Max slope = 1.488–0.009*age	−0.017, −0.0002	0.045	−2.03	0.04	−0.23	0.72 (0.55–0.87)
PeEn = 1.880 + 0.002*age	0.001, 0.004	0.008	2.72	0.07	0.15	0.27 (0.12 0.44)
log(varBS) = 6.03–0.03*age	−0.05, −0.02	<0.001	−3.90	0.13	−0.33	0.85 (0.71–0.95)

*CI, confidence interval; rho, Spearman’s correlation coefficient*.

### Time-Domain Analyses

**Figure 4:**
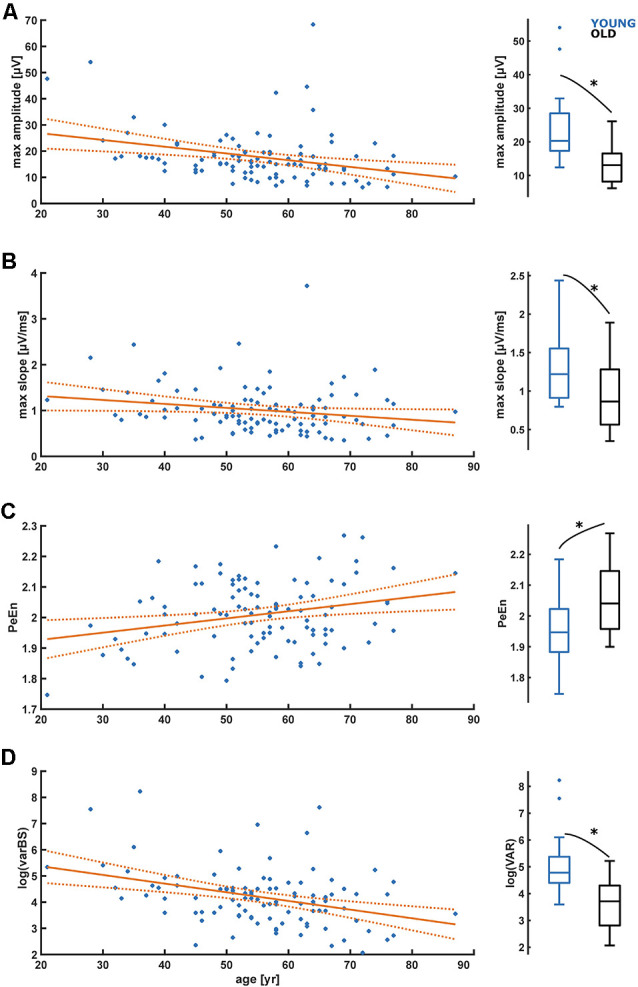
Linear regression models regarding the influence of age on maximum amplitude **(A)**, maximum slopes **(B)**, permutation entropy **(C)**, and signal variance in burst suppression **(D)**. **(A)** The maximum amplitude significantly (*p* = 0.001) decreases with age. **(B)** The maximum slope significantly (*p* = 0.045) decreases with age. **(C)** Maximum permutation entropy (PeEn) significantly (*p* = 0.008) increases with age. **(D)** Signal variance in burst suppression (varBS) significantly (*p* < 0.001) decreases with age. *Indicates a significant difference (*p* < 0.05).

For the time domain analyses, we observed a significant decrease in amplitude (*p* = 0.001) and maximum slope (*p* = 0.045) with age as presented in Figures 4A,B as well as in Table 1. We further found a significant increase (*p* = 0.008) in PeEn with age (Figure 4C). For all patients included, we found an “excellent” separation in the maximum EEG amplitudes [YOUNG: 20.29 (12.40–54.00) μV; OLD: 13.07 (6.20–26.11) μV; AUC = 0.84 (0.69 0.95)] and an “acceptable” separation for the maximum slopes [YOUNG: 1.22 (0.80 2.44) μV/ms; OLD: 0.86 (0.35–1.89) μV/ms; AUC = 0.72 (0.55 0.87)]. The PeEn was higher in the OLD [2.04 (1.90–2.27)] than in the YOUNG [1.94 (1.75–2.18); AUC = 0.73 (0.56 0.88)]. The separation was acceptable. We also found a significant decrease (*p* < 0.001) in the local EEG variance (see “Materials and Methods” section) *varBS* with age (Figure 4D). The separation between YOUNG and OLD was “excellent” with an AUC = 0.85 (0.70 0.95).

### Sub-analyses Regarding a Possible Impact of Midazolam

Eighty-three out of 102 patients received pre-operative midazolam. To examine a potential contribution of midazolam pre-medication to our observation of age-related changes in burst suppression, we applied our linear regression model to a subset of data including the patients with midazolam pre-medication only and found similar results. Age-related decreases in alpha band power (*p* < 0.001), EEG amplitude (*p* = 0.003) and maximum EEG slope (*p* = 0.019) persisted in this subset, and PeEn increased (*p* = 0.033). The plots for the linear models are presented in the Supplementary Figure 5 and the coefficients are presented in Supplementary Table 1. Examination of the binary variables YOUNG vs. OLD showed a similar trend, but we did not observe significant differences in the normalized power in the high frequencies. Supplementary Figure 6 presents the corresponding (n)PSD plots and statistical results. The separation of alpha power was “excellent,” with lower alpha power in the premedicated OLD [AUC = 0.90 (0.75 1)]. We found no effect of age when comparing YOUNG and premedicated OLD for the relative alpha power [AUC = 0.63 (0.42 0.82)] and SpEnt [AUC = 0.39 (0.19 0.61)], but in premedicated OLD, maximum EEG amplitudes were lower [AUC = 0.89 (0.75 1), “excellent” effect] and the maximum slope was flatter [AUC = 0.77 (0.58 0.92), “acceptable” effect]. PeEn was higher in the premedicated OLD in a [AUC = 0.30 (0.13 0.50), “acceptable” effect]. Because only 6/25 patients in the OLD group received midazolam we are aware of the small sample size that presents a limitation of the investigation. Further research will be necessary to give a definite answer.

Interestingly, although the sample size is small, we observed differences in the spectral composition of the EEG between the OLD patients that did receive midazolam and OLD patients that did not receive midazolam. Non-premedicated OLD patients had more high-frequency contents in their burst EEG as presented in Supplementary Figure 7.

Although it remains possible that midazolam has an influence on burst characteristics in this population, we are reluctant to draw this conclusion as many factors influence the decision to administer midazolam (e.g., subjective assessment of cognitive frailty).

## Discussion

Here we demonstrate an influence of age on the burst characteristics during anesthetic-induced EEG burst suppression. We found that the spectral EEG characteristics of the initial burst changed with age. Older patients had less power in the low frequencies up to ~15 Hz. When normalizing the power spectrum, we could observe a higher contribution of the higher frequencies starting at around 15 Hz in the old patients to the total power. Similar changes with age in total and relative power were shown for the EEG under general anesthesia without burst suppression (Schultz et al., 2004; Purdon et al., 2015; Kreuzer et al., 2020). Consequently, older patients had lower amplitudes and flatter EEG slopes that led to a lower EEG power in the alpha band as well as to a higher signal irregularity as reflected by the higher PeEn. Because we focused on the initial burst, i.e., at the state change of the brain from the oscillatory to the burst suppression mode, the observed differences are not due to a different “state of burst suppression” as a result of a pharmacologic effect. We decided to not focus on the concentration, but on the time point of the state change in EEG activity to ensure comparability of our recordings. When considering only patients pre-medicated with midazolam the results were very similar.

Although it is known that total EEG power decreases with age for other quiescent states like general anesthesia without BSUPP (Schultz et al., 2004; Purdon et al., 2015; Kreuzer et al., 2020), sleep (Carrier et al., 2001), or wakefulness (Polich, 1997), this is definitive evidence that the burst amplitude also decreases with age. While this is not surprising, this finding may have implications for the use and design of processed-EEG monitoring approaches. Current monitoring systems such as the bispectral index (Rampil, 1998), the patient state index (Drover et al., 2002), or the state and response entropy (Viertio-Oja et al., 2004) calculate the (burst) suppression ratio (BSR) to display the occurrence and strength of BSUPP. The BSR reflects the proportion of suppressed EEG within a defined period, for instance, 63 s for the bispectral index (Muhlhofer et al., 2017). Most BSR algorithms focus on the detection of suppressed EEG as evidence for a BSUPP episode. If the EEG amplitude is below a set threshold for a defined duration, BSUPP is detected (Rampil, 1998; Särkelä et al., 2002; Jensen et al., 2014). Artifacts (i.e., EKG or motion) for instance can contaminate the signal and can spuriously cause increased EEG activity, thus hindering algorithms that detect BSUPP by identifying suppression (Willingham and Avidan, 2017). This leads to an underestimation of BSUPP by the monitoring devices (Muhlhofer et al., 2017) and to a possible misclassification of the BSUPP EEG as an awake EEG (Hart et al., 2009; Willingham and Avidan, 2017). Other reported approaches to detect BSUPP based on signal variance (An et al., 2015), may prove superior to commercially used algorithms but they also focus on the suppression episode or the transition from suppression to burst. In order to detect suppression a threshold variance value is defined and then a logical decision is made. If the local variance is below the threshold the EEG segment is considered to be suppression. Since age reduces the local variance during bursts, adjustment of the threshold is necessary to not increase the amount of detected suppression.

Hence, EEG information derived during the bursts could at least function as a quality and plausibility check for BSUPP detection. But to correctly interpret the information from the burst, the parameter should be corrected for age.

Again, we focused on the first identifiable burst and did not consider any propofol concentrations for detection, because first, older patients require less propofol, and second, because BSUPP can also occur at very low anesthetic concentrations as described by Sessler et al. (2012).

One may question the relevance of reliable BSUPP detection, but only if there are tools that can reliably detect burst suppression in all patients we as researchers may be able to investigate the proposed investigation of burst suppression and an increased risk of postoperative neurocognitive disorders. Currently, there is an ongoing, controversial discussion regarding the association of postoperative neurocognitive disorders with *excessive doses of hypnotic agents* and/or BSUPP. Several studies highlighted a possible association between this intraoperative EEG pattern and delirium in the postoperative care unit (Hesse et al., 2019) or postoperative delirium (Soehle et al., 2015; Fritz et al., 2016). Other studies could not show this relationship (Shortal et al., 2019; Wildes et al., 2019). In particular, the study by Shortal et al. shows that young volunteers may not be affected by BSUPP (Shortal et al., 2019). But these studies use different tools and criteria to identify burst suppression. Some results for instance were derived from processed EEG information only (Soehle et al., 2015), while others used spectral EEG representations (Shortal et al., 2019) or the raw EEG (Wildes et al., 2019) for burst suppression identification. Because the processed EEG burst suppression indices may underestimate the real occurrence of burst suppression (Muhlhofer et al., 2017) the studies using these indices may not be easily compared to other studies. Hence, standardized burst suppression detection may prove helpful. And the inclusion of the EEG burst information in an age-adjusted manner could help to achieve this goal.

## Limitations

Of course, our approach has some limitations. First of all, we only describe changes happening in frontal regions of the cortex, and to optimize burst suppression detection, the analysis of spatiotemporal dynamics over the entire cortex could be useful. Earlier findings described spatiotemporal differences in BSUPP features (Lewis et al., 2013). Nevertheless, as a consequence of feasibility, current monitoring devices usually record the patient’s EEG from the forehead. Hence, we chose the frontal EEG for our research approach. Further, the effects described are only for propofol-induced bursts and the results could be different for other anesthetics since the choice of the anesthetic regimen, for instance, influences the burst characteristics (Kenny et al., 2014; Fleischmann et al., 2018). Also, the role of ischemic events that may be related to the development of burst suppression (Morimoto et al., 2005; Kertai et al., 2011) will have to be investigated more closely in the future to distinguish them from drug-induced burst suppression. While we could describe an age-related change in the EEG burst characteristics we did not control for any confounders such as comorbidities that come with age. This needs to be done with a properly sized data set and an appropriate study design. A part of our patient collective, and especially the younger patients, received midazolam as premedication. Midazolam is a potent agonist of the γ-aminobutyric acid receptor type A (GABA_A_ receptor) and premedication with midazolam seems to modify intraoperative EEG signatures (Windmann et al., 2019). As far as we can interpret our results from the limited sample size, we observed similar results as for the analyses of the entire data set. But one should have in mind that the sample size is underpowered for this observation. Still, our data indicate that midazolam premedication may reduce high-frequency brain activity within the bursts in old patients. Midazolam as well as propofol have been demonstrated to promote low-frequency oscillations in the brain (Yeom et al., 2017). Mechanistically midazolam additively or synergistically interacts with propofol depending on the GABA concentration (McAdam et al., 1998) and might therefore further enhance cortical inhibition finally resulting in a less high-frequency activity.

## Conclusion

Age-related EEG features identified for general anesthesia are also present during burst suppression in the architecture of the bursts. The burst amplitude decreases and the bursts become more irregular. As a consequence, automated detection approaches for BSUPP may become less accurate with age. Our findings highlight the necessity of age-adjusted monitoring approaches to optimize monitoring for old patients.

## Data Availability Statement

The original contributions presented in the study are included in the article/supplementary material, further inquiries can be directed to the corresponding author. Requests to access these datasets should be directed to MK, m.kreuzer@tum.de.

## Ethics Statement

The studies involving human participants were reviewed and approved by local Ethics or Institutional Review Boards (Emory University). The patients/participants provided their written informed consent to participate in this study.

## Author Contributions

PSG collected the data. MK and SK designed the analyses. DPO, MS, and MK analyzed the data. DPO, MS, GS, PSG, SK, and MK discussed the results and helped to write the manuscript. All authors contributed to the article and approved the submitted version.

## Conflict of Interest

The authors declare that the research was conducted in the absence of any commercial or financial relationships that could be construed as a potential conflict of interest.

## References

[B1] An J. Jonnalagadda D. Moura V. Purdon P. L. Brown E. N. Westover M. B. (2015). “Spatial variation in automated burst suppression detection in pharmacologically induced coma,” 37th Annual International Conference of the IEEE Engineering in Medicine and Biology Society (EMBC), (Milan: IEEE), 7430–7433. 10.1109/EMBC.2015.7320109 PMC4876722 26738009

[B3] Bandt C. Pompe B. (2002). Permutation entropy: a natural complexity measure for time series. Phys. Rev. Lett. 88:174102. 10.1103/PhysRevLett.88.174102 12005759

[B4] Berger M. Mark J. B. Kreuzer M. (2020). Of parachutes, speedometers and EEG: what evidence do we need to use devices and monitors? Anesth. Analg. 130, 1274–1277. 10.1213/ANE.0000000000004653 32287134 PMC7735682

[B5] Berger S. Schneider G. Kochs E. F. Jordan D. (2017). Permutation entropy: too complex a measure for EEG time series? Entropy 19:692 10.3390/e19120692

[B6] Brown E. N. Lydic R. Schiff N. D. (2010). General anesthesia, sleep and coma. N. Engl. J. Med. 363, 2638–2650. 10.1056/NEJMra0808281 21190458 PMC3162622

[B7] Carrier J. Land S. Buysse D. J. Kupfer D. J. Monk T. H. (2001). The effects of age and gender on sleep EEG power spectral density in the middle years of life (ages 20-60 years old). Psychophysiology 38, 232–242. 10.1017/S0048577201991838 11347869

[B8] Drover D. R. Lemmens H. J. Pierce E. T. Plourde G. Loyd G. Ornstein E.. (2002). Patient state index: titration of delivery and recovery from propofol, alfentanil and nitrous oxide anesthesia. Anesthesiology 97, 82–89. 10.1097/00000542-200207000-00012 12131107

[B9] Fleischmann A. Pilge S. Kiel T. Kratzer S. Schneider G. Kreuzer M. (2018). Substance-specific differences in human electroencephalographic burst suppression patterns. Front. Hum. Neurosci. 12:368. 10.3389/fnhum.2018.00368 30297992 PMC6160564

[B10] Fritz B. A. Kalarickal P. L. Maybrier H. R. Muench M. R. Dearth D. Chen Y.. (2016). Intraoperative electroencephalogram suppression predicts postoperative delirium. Anesth. Analg. 122, 234–242. 10.1213/ANE.0000000000000989 26418126 PMC4684753

[B11] García P. S. (2020). Meta-analysis and megadata in electroencephalogram-based techniques for delirium prevention. Anesth. Analg. 131, 709–711. 10.1213/ANE.0000000000004867 32940441

[B12] Viertio-Oja H. Maja V. Sarkela M. Talja P. Tenkanen N. Tolvanen-Laakso H.. (2004). Description of the Entropy algorithm as applied in the Datex-Ohmeda a/5 Entropy Module. Acta. Anaesthesiol. Scand. 48, 154–161. 10.1111/j.0001-5172.2004.00322.x 14995936

[B13] Hart S. M. Buchannan C. R. Sleigh J. W. (2009). A failure of M-entropy to correctly detect burst suppression leading to sevoflurane overdosage. Anaesth. Intensive Care 37, 1002–1004. 10.1177/0310057X0903700619 20014609

[B14] Hentschke H. Stüttgen M. C. (2011). Computation of measures of effect size for neuroscience data sets. Eur. J. Neurosci. 34, 1887–1894. 10.1111/j.1460-9568.2011.07902.x 22082031

[B15] Hesse S. Kreuzer M. Hight D. Gaskell A. Devari P. Singh D.. (2019). Association of electroencephalogram trajectories during emergence from anaesthesia with delirium in the post-anaesthesia care unit: an early sign of postoperative complications. Br. J. Anaesth. 122, 622–634. 10.1016/j.bja.2018.09.016 30915984 PMC6465086

[B16] Jensen E. W. Valencia J. F. Lopez A. Anglada T. Agusti M. Ramos Y.. (2014). Monitoring hypnotic effect and nociception with two EEG-derived indices, qCON and qNOX, during general anaesthesia. Acta Anaesthesiol. Scand. 58, 933–941. 10.1111/aas.12359 24995461

[B17] Kenny J. D. Westover M. B. Ching S. Brown E. N. Solt K. (2014). Propofol and sevoflurane induce distinct burst suppression patterns in rats. Front. Syst. Neurosci. 8:237. 10.3389/fnsys.2014.00237 25565990 PMC4270179

[B18] Kertai M. D. Palanca B. J. Pal N. Burnside B. A. Zhang L. Sadiq F.. (2011). Bispectral index monitoring, duration of bispectral index below 45, patient risk factors and intermediate-term mortality after noncardiac surgery in the B-unaware trial. Anesthesiology 114, 545–556. 10.1097/ALN.0b013e31820c2b57 21293252

[B19] Kreuzer M. Stern M. A. Hight D. Berger S. Schneider G. Sleigh J. W.. (2020). Spectral and entropic features are altered by age in the electroencephalogram in patients under sevoflurane anesthesia. Anesthesiology 132, 1003–1016. 10.1097/ALN.0000000000003182 32108685 PMC7159998

[B20] Lewis L. D. Ching S. Weiner V. S. Peterfreund R. A. Eskandar E. N. Cash S. S.. (2013). Local cortical dynamics of burst suppression in the anaesthetized brain. Brain 136, 2727–2737. 10.1093/brain/awt174 23887187 PMC3754454

[B21] Lukatch H. S. Kiddoo C. E. Maciver M. B. (2005). Anesthetic-induced burst suppression EEG activity requires glutamate-mediated excitatory synaptic transmission. Cereb. Cortex 15, 1322–1331. 10.1093/cercor/bhi015 15647528

[B22] Mandrekar J. N. (2010). Receiver operating characteristic curve in diagnostic test assessment. J. Thorac. Oncol. 5, 1315–1316. 10.1097/JTO.0b013e3181ec173d 20736804

[B300] Mayhew D. Mendonca V. Murthy B. V. S. (2019). A review of ASA physical status—historical perspectives and modern developments. Anaesthesia 74, 373–379. 30648259 10.1111/anae.14569

[B23] McAdam L. C. MacDonald J. F. Orser B. A. (1998). Isobolographic analysis of the interactions between midazolam and propofol at GABA(A) receptors in embryonic mouse neurons. Anesthesiology 89, 1444–1454. 10.1097/00000542-199812000-00022 9856719

[B24] McDonald J. H. (2009). Handbook of Biological Statistics. Baltimore, MD: Sparky House Publishing.

[B25] Morimoto Y. Matsumoto A. Koizumi Y. Gohara T. Sakabe T. Hagihira S. (2005). Changes in the bispectral index during intraabdominal irrigation in patients anesthetized with nitrous oxide and sevoflurane. Anesth. Analg. 100, 1370–1374. 10.1213/01.ANE.0000148124.02288.D1 15845688

[B26] Muhlhofer W. Zak R. Kamal T. Rizvi B. Sands L. Yuan M.. (2017). Burst-suppression ratio underestimates absolute duration of electroencephalogram suppression compared with visual analysis of intraoperative electroencephalogram. Br. J. Anaesth. 118, 755–761. 10.1093/bja/aex054 28486575 PMC6224027

[B27] Pilge S. Jordan D. Kreuzer M. Kochs E. F. Schneider G. (2014). Burst suppression-MAC and burst suppression-CP_50_ as measures of cerebral effects of anaesthetics. Br. J. Anaesth. 112, 1067–1074. 10.1093/bja/aeu016 24658022

[B28] Polich J. (1997). EEG and ERP assessment of normal aging. Electroencephalogr. Clin. Neurophysiol. 104, 244–256. 10.1016/s0168-5597(97)96139-6 9186239

[B29] Purdon P. Pavone K. Akeju O. Smith A. Sampson A. Lee J.. (2015). The ageing brain: age-dependent changes in the electroencephalogram during propofol and sevoflurane general anaesthesia. Br. J. Anaesth. 115, i46–i57. 10.1093/bja/aev213 26174300 PMC4501918

[B30] Rampil I. J. (1998). A primer for EEG signal processing in anesthesia. Anesthesiology 89, 980–1002. 10.1097/00000542-199810000-00023 9778016

[B31] Särkelä M. Mustola S. Seppänen T. Koskinen M. Lepola P. Suominen K.. (2002). Automatic analysis and monitoring of burst suppression in anesthesia. J. Clin. Monit. Comput. 17, 125–134. 10.1023/a:1016393904439 12212991

[B32] Schultz A. Grouven U. Zander I. Beger F. A. Siedenberg M. Schultz B. (2004). Age-related effects in the EEG during propofol anaesthesia. Acta. Anaesthesiol. Scand. 48, 27–34. 10.1111/j.1399-6576.2004.00258.x 14674970

[B33] Sessler D. I. Sigl J. C. Kelley S. D. Chamoun N. G. Manberg P. J. Saager L.. (2012). Hospital stay and mortality are increased in patients having a “triple low” of low blood pressure, low bispectral index and low minimum alveolar concentration of volatile anesthesia. Anesthesiology 116, 1195–1203. 10.1097/ALN.0b013e31825683dc 22546967

[B34] Shortal B. P. Hickman L. B. Mak-McCully R. A. Wang W. Brennan C. Ung H.. (2019). Duration of EEG suppression does not predict recovery time or degree of cognitive impairment after general anaesthesia in human volunteers. Br. J. Anaesth. 123, 206–218. 10.1016/j.bja.2019.03.046 31202561 PMC6676227

[B35] Soehle M. Dittmann A. Ellerkmann R. K. Baumgarten G. Putensen C. Guenther U. (2015). Intraoperative burst suppression is associated with postoperative delirium following cardiac surgery: a prospective, observational study. BMC Anesthesiol. 15:61. 10.1186/s12871-015-0051-7 25928189 PMC4419445

[B36] Swank R. L. Watson C. W. (1949). Effects of barbiturates and ether on spontaneous electrical activity of dog brain. J. Neurophysiol. 12, 137–160. 10.1152/jn.1949.12.2.137 18114367

[B37] Wildes T. S. Mickle A. M. Ben Abdallah A. Maybrier H. R. Oberhaus J. Budelier T. P.. (2019). Effect of electroencephalography-guided anesthetic administration on postoperative delirium among older adults undergoing major surgery: the ENGAGES randomized clinical trial. JAMA 321, 473–483. 10.1001/jama.2018.22005 30721296 PMC6439616

[B38] Willingham M. D. Avidan M. S. (2017). Triple low, double low: it’s time to deal Achilles heel a single deadly blow. Br. J. Anaesth. 119, 1–4. 10.1093/bja/aex132 28575210

[B39] Windmann V. Spies C. Brown E. N. Kishnan D. Lichtner G. Koch S.. (2019). Influence of midazolam premedication on intraoperative EEG signatures in elderly patients. Clin. Neurophysiol. 130, 1673–1681. 10.1016/j.clinph.2019.05.035 31351371

[B40] Yan R. Liu Y. Gao R. X. (2012). Permutation entropy: a nonlinear statistical measure for status characterization of rotary machines. Mech. Syst. Signal Process. 29, 474–484. 10.1016/j.ymssp.2011.11.022

[B41] Yeom S.-K. Won D.-O. Chi S. I. Seo K.-S. Kim H. J. Müller K.-R.. (2017). Spatio-temporal dynamics of multimodal EEG-fNIRS signals in the loss and recovery of consciousness under sedation using midazolam and propofol. PLoS One 12:e0187743. 10.1371/journal.pone.0187743 29121108 PMC5679575

