# Supplementary material for: Age-Related EEG Features of Bursting Activity During Anesthetic-Induced Burst Suppression
Source: Front Syst Neurosci. 2020 Dec 3;14:599962. doi: 10.3389/fnsys.2020.599962 (PMC7744408; doi:10.3389/fnsys.2020.599962)
Supplement: Supplementary file 1 [file Data_Sheet_1.docx]

# *Supplemental Information for*

# *Age-Related EEG features of Bursting Activity During Anesthetic-Induced Burst Suppression*

**
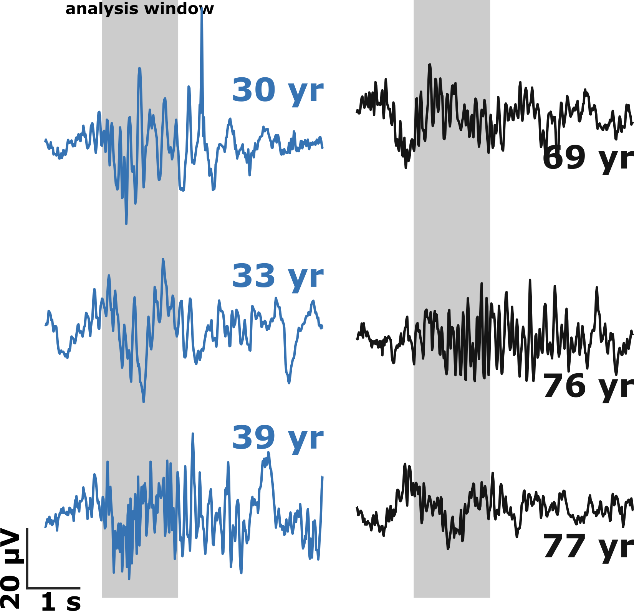
**

**Figure S1 presents exemplary initial bursts for patients from the OLD and YOUNG group.**


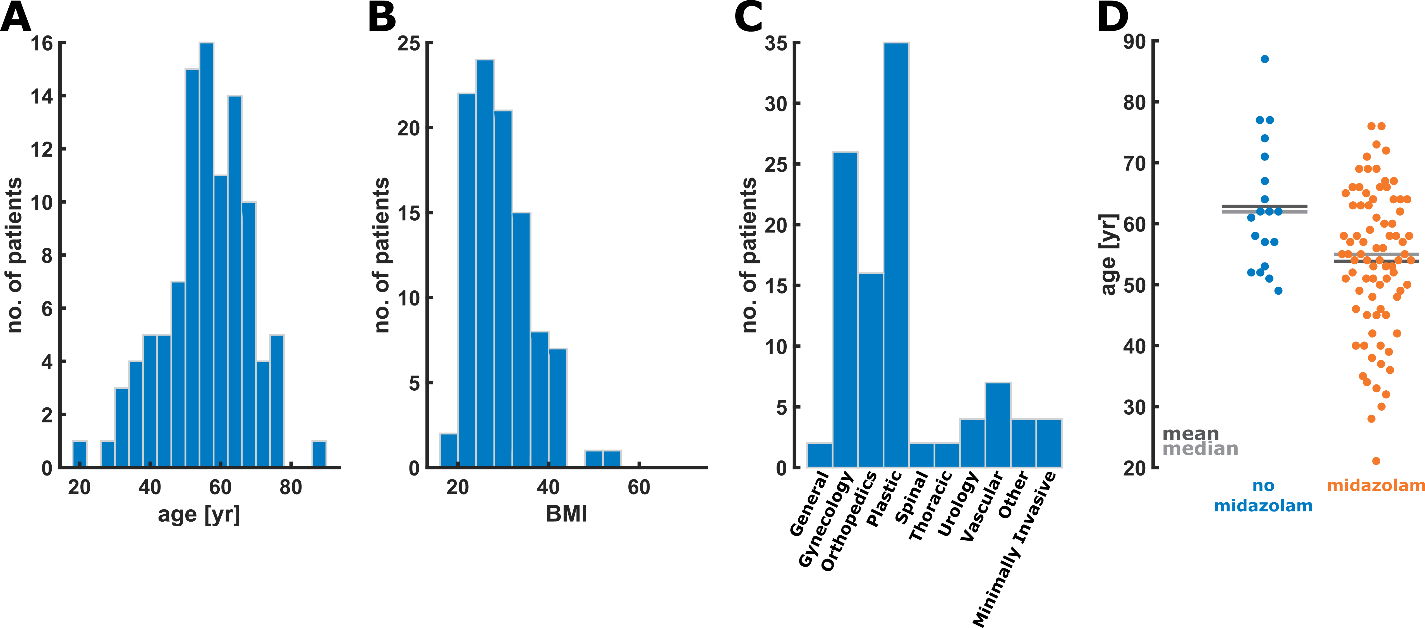


**Figure S2: Histogram and beeswarm plots of the demographic information.**

1. **Age distribution among the patients**
2. **Body mass index (BMI) distribution among the patients**
3. **Interventions separated by specialty**
4. **Age distribution of patients that either did or did not receive midazolam**


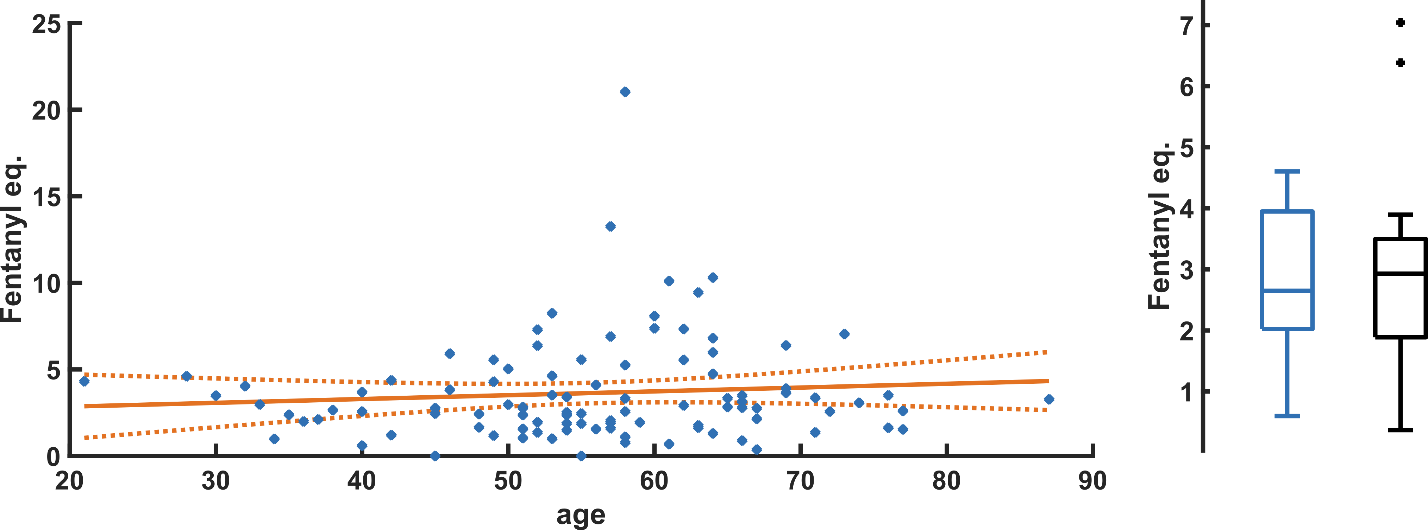


**Figure S3: We did not observe a significant (linear) change of the fentanyl equivalents with age. *fentanyl equivalents µgkg^-1^ = 2.41+0.02*age* (p=0.383; R^2^=0.00). When comparing the YOUNG (blue) vs. OLD (black) group, the AUC with 95% Ci was 0.49 [0.29 0.69].**

**
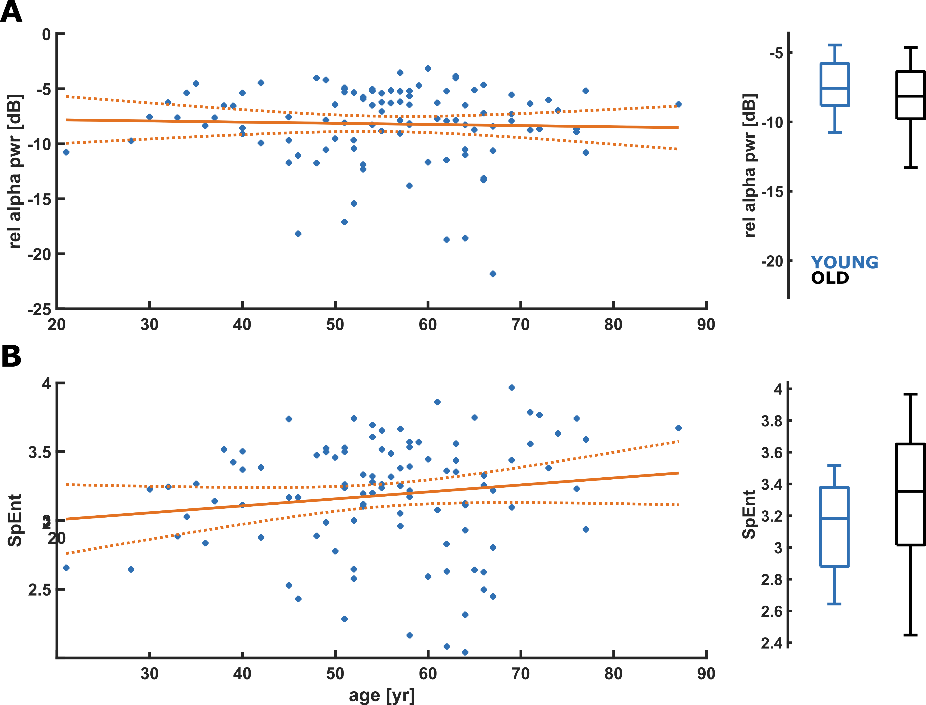
**

**Figure S4: Linear model between relative alpha power (A) and spectral entropy (SpEnt) (B) with age together with the corresponding box plots comparing the OLD versus the YOUNG group.**

1. **There was no significant change in relative alpha power with age: *rel. alpha pwr=-0.01*age-7.60* (p=0.719); YOUNG vs. OLD: AUC=0.60 [0.40 0.78]**

**There was no significant change in spectral entropy with age; *SpEnt=0.01*age+2.90* (p=0.145); YOUNG vs. OLD: AUC=0.36 [0.18 0.55]**


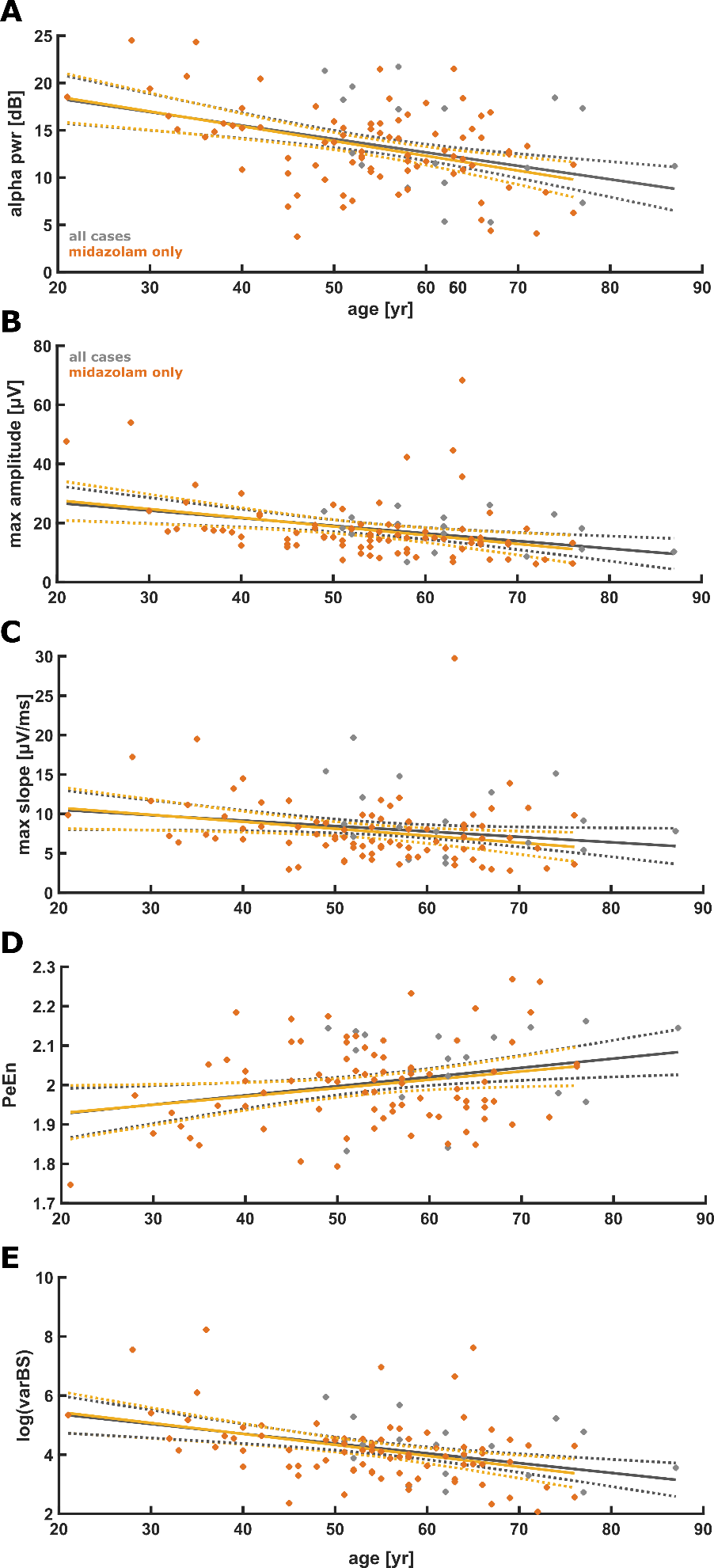


**Figure S5: Linear regression models for all patients (grey) and patients only that received midazolam (orange) regarding the influence of age on alpha power (A) maximum amplitude (B), maximum slopes (C), permutation entropy (D), and signal variance in burst suppression (E). As for all patients we found similar results for the midazolam patients.**

1. **Alpha power significantly (p<0.001) decreases with age.**
2. **Maximum amplitude significantly (p=0.003) decreases with age.**
3. **Maximum slope significantly (p=0.019) decreases with age.**
4. **Maximum permutation entropy (PeEn) significantly (p=0.033) increases with age.**
5. **Signal variance in burst suppression (varBS) significantly (p<0.001) decreases with age.**

**
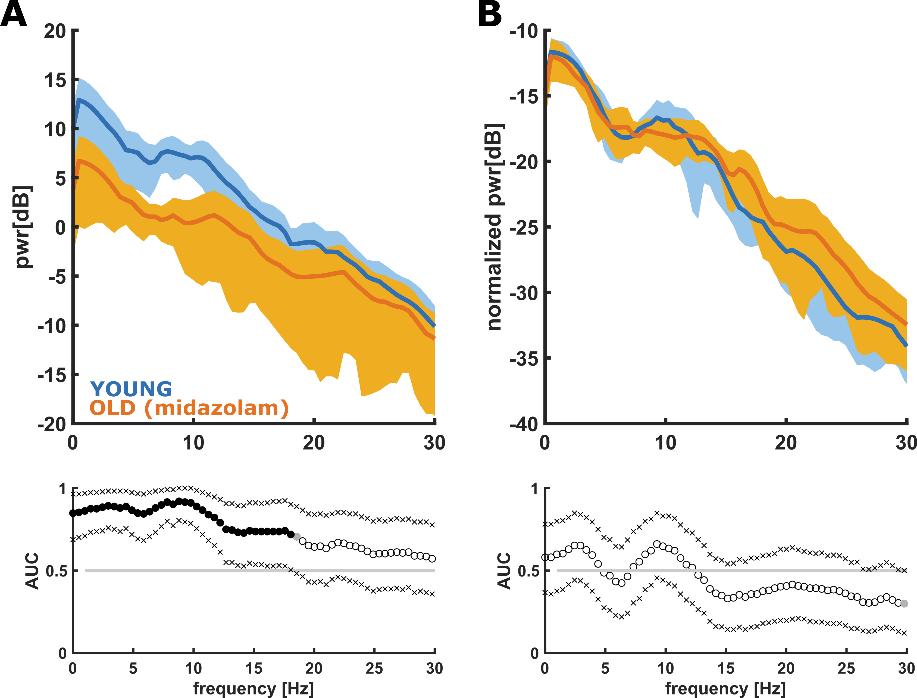
**

**Figure S6: Absolute (A) and normalized (B) power spectral density and corresponding AUC with 95% confidence intervals for the young (<45 years) and old (>65 years) patients that received midazolam.**

1. **YOUNG patients had significantly higher power in the frequencies up to ~18 Hz.**
2. **There were no significant differences in normalized alpha power between the groups.**

| **only patients receiving midazolam** |  |  | **YOUNG vs OLD (AUC)** |
| --- | --- | --- | --- |
| *alpha power = 21.67-0.16*age* | <0.001 | 0.18 | 0.90 [0.75 1] |
| *normalized alpha power = -8.11-0.00*age* | 0.998 | 0 | 0.63 [0.42 0.82] |
| *SpEnt = 2.91+0.00*age* | 0.209 | 0.02 | 0.39 [0.19 0.61] |
| *max amplitude* *= 33.60-0.29*age* | 0.003 | 0.11 | 0.89 [0.75 1] |
| *max slope* *= 1.568-0.011*age* | 0.019 | 0.07 | 0.77 [0.58 0.92] |
| *PeEn* *= 1.89+0.002*age* | 0.033 | 0.05 | 0.30 [0.13 0.50] |
| log(*varBS)* *= 6.18-0.04*age* | <0.001 | 0.15 | 0.88 [0.75 0.98] |
|  |  |  |  |

**Table S1: Statistical parameters of the linear model (EEG parameter vs. age) for the patient cohort that received midazolam**

**
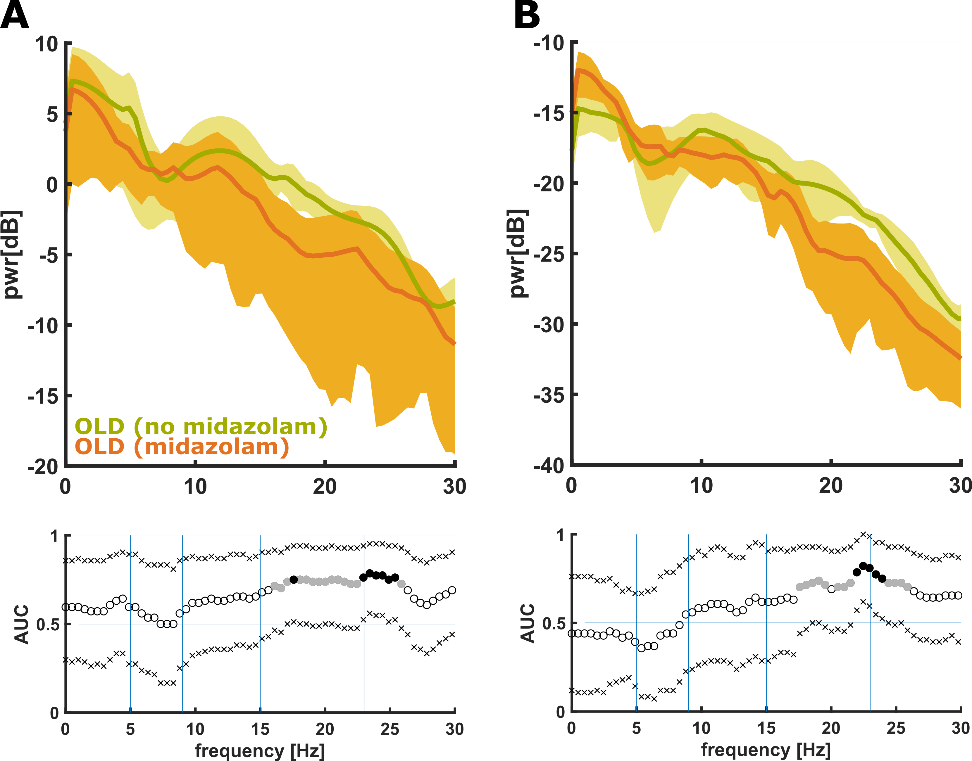
**

**Figure S7: Absolute (A) and normalized (B) power spectral density and corresponding AUC with 95% confidence intervals for the old (>65 years) patients that did (orange) or did not (green) receive midazolam.**

1. **Patients, premedicated with midazolam had significantly lower power in the range from ~17-25 Hz.**
2. **Patients, premedicated with midazolam had significantly lower normalized power in the range from ~17-25 Hz.**
